# The role of peripheral inflammatory insults in Alzheimer’s disease: a review and research roadmap

**DOI:** 10.1186/s13024-023-00627-2

**Published:** 2023-06-05

**Authors:** Keenan A. Walker, Lydia M. Le Page, Niccolò Terrando, Michael R. Duggan, Michael T. Heneka, Brianne M. Bettcher

**Affiliations:** 1 Laboratory of Behavioral Neuroscience, National Institute On Aging. Baltimore, Baltimore, MD USA; 2 Departments of Physical Therapy and Rehabilitation Science, and Radiology and Biomedical Imaging, University of California San Francisco, San Francisco, CA USA; 3 Department of Anesthesiology, Cell Biology and Immunology, Duke University Medical Center, Durham, NC USA; 4 Luxembourg Centre for Systems Biomedicine, University of Luxembourg, Belvaux, Luxembourg; 5 Behavioral Neurology Section, Department of Neurology, University of Colorado Alzheimer’s and Cognition Center, University of Colorado Anschutz Medical Campus, Aurora, CO USA

**Keywords:** Dementia, Inflammation, Neuroinflammation, Systemic inflammation, Peripheral inflammation, Infection, Neuro-immune axis, Cytokines

## Abstract

Peripheral inflammation, defined as inflammation that occurs outside the central nervous system, is an age-related phenomenon that has been identified as a risk factor for Alzheimer’s disease. While the role of chronic peripheral inflammation has been well characterized in the context of dementia and other age-related conditions, less is known about the neurologic contribution of acute inflammatory insults that take place outside the central nervous system. Herein, we define acute inflammatory insults as an immune challenge in the form of pathogen exposure (e.g., viral infection) or tissue damage (e.g., surgery) that causes a large, yet time-limited, inflammatory response. We provide an overview of the clinical and translational research that has examined the connection between acute inflammatory insults and Alzheimer’s disease, focusing on three categories of peripheral inflammatory insults that have received considerable attention in recent years: acute infection, critical illness, and surgery. Additionally, we review immune and neurobiological mechanisms which facilitate the neural response to acute inflammation and discuss the potential role of the blood–brain barrier and other components of the neuro-immune axis in Alzheimer’s disease. After highlighting the knowledge gaps in this area of research, we propose a roadmap to address methodological challenges, suboptimal study design, and paucity of transdisciplinary research efforts that have thus far limited our understanding of how pathogen- and damage-mediated inflammatory insults may contribute to Alzheimer’s disease. Finally, we discuss how therapeutic approaches designed to promote the resolution of inflammation may be used following acute inflammatory insults to preserve brain health and limit progression of neurodegenerative pathology.

**Supplementary Information:**

The online version contains supplementary material available at 10.1186/s13024-023-00627-2.

## Background

Alzheimer’s disease (AD), a neurodegenerative condition that affects approximately 24 million people worldwide, accounts for 60 to 70% of all dementia cases [1]. As lifespan increases and more people live into the 7^th^, 8^th^, and 9^th^ decades of life, the prevalence of AD is expected to increase to 70 million by 2030 [2]. Despite tremendous recent advancements in neurodegenerative disease research, the understanding of AD biology remains incomplete. Although amyloid-beta (Aβ) plaques and tau neurofibrillary tangles are considered hallmark features of AD, the past two decades have seen a surge in genomic studies that consistently point to the central role of microglia and neuro-immune dysfunction in the pathogenesis of AD. Concurrently, a large body of work has highlighted the potential relevance of peripheral immune changes, particularly pro-inflammatory signaling, in AD pathogenesis. Evidence for a relationship between peripheral immune factors and AD has come primarily from epidemiological and observational research studies which demonstrate associations between circulating inflammatory markers and neurocognitive features. However, there is a growing body of literature supporting the role of acute inflammatory insults as potential catalysts for cognitive decline and AD. Here, we define *acute inflammatory insult* as an immune challenge – typically tissue injury or exposure to a pathogen – that produces, in most cases, a time-limited inflammatory response.

This review focuses on the evidence from clinical and translational research linking acute inflammatory insults to cognitive decline and AD. We review evidence for the role of both pathogen- and damage-mediated inflammatory insults in AD and provide current conceptualizations of mechanisms for and treatment of immune-mediated cognitive decline. Importantly, we outline critical next steps for understanding how acute inflammatory insults might impact AD pathological processes and influence clinical presentation. We provide guidelines to address gaps in the literature, outline methods for appraisal of infection-mediated outcomes, and highlight future studies that may accelerate therapeutic interventions. A comprehensive review of systemic inflammation, vaccines, immune-related biomarkers, and chronic infection in the context of AD is outside the scope of this paper. These topics have been reviewed extensively elsewhere [3–6].

### Peripheral inflammation in Alzheimer’s disease

Peripheral inflammation, defined here as inflammation occurring outside the central nervous system, has been described as a potential risk factor for AD and vascular dementia [7, 8]. Recent meta-analyses of 170 + case–control studies found that inflammatory proteins such as IL-1β, IL-6, IL-18, sTNFR1, IFN-γ, high-sensitivity C-reactive protein (CRP), and α1-antichymotrypsin are elevated in the blood of AD patients, compared to that of neurologically normal individuals [8, 9]. The results of these cross-sectional analyses have been supported by a series of cohort studies which demonstrate that cognitively normal individuals with elevated inflammatory markers in blood are at greater risk for future mild cognitive impairment (MCI), AD dementia, and all-cause dementia [10–14]. Importantly, the association between inflammatory proteins and neurocognitive outcomes varies based on the molecule in question – that is, distinct inflammatory proteins vary widely in terms of their relationships with brain structure, function, and AD risk [8, 9, 15]. The relationship between inflammatory proteins and AD-related outcomes also varies by disease stage [7]. Some inflammatory proteins are elevated in the asymptomatic phase of AD, whereas others may only become abnormal when individuals develop MCI or dementia [4]. Importantly, elevations in several inflammatory cytokines, such as IFN-γ and IL-12p70, which influence Th1-macrophage activation, have shown a protective effect on AD-related neurocognitive outcomes [16]. Such findings underscore the need to further understand how disease stage, underlying pathology, and the specific inflammatory molecule in question may influence the relationship between inflammatory proteins and dementia risk. Given that there are hundreds of secreted immune and inflammatory proteins in circulation, analyses of the larger inflammatory proteome, alongside pathway and network analyses, will likely be needed to yield more specific insights about the immune pathway- and disease stage-specific role of inflammatory proteins in the decades leading up to AD dementia [17].

The studies reviewed above have focused almost exclusively on understanding the link between *low-grade inflammation* and AD or related outcomes. Low-grade inflammation can be characterized by modest elevations in circulating inflammatory proteins, which can occur as a result of clinical or subclinical disease processes, age-related changes in biology (e.g., cellular senescence), or for some, without any obvious antecedent [18]. Whereas inflammation is an important and necessary feature of the immune response, it is known to be potentially harmful in the context of chronic activation. Several studies have demonstrated that individuals who maintain elevated or show increasing inflammatory proteins across time tend to have the poorest brain-related outcomes [19–21]. Although the link between early/chronic peripheral inflammation and AD-related outcomes provides some temporal support for a mechanistic role of inflammatory proteins in AD pathogenesis, the possibility that these molecules represent a response to, rather than a cause of, AD pathophysiology cannot be ruled out. Mendelian randomization analyses have so far provided mixed support for the causal role of peripheral inflammatory proteins in AD [22–26]. Nonetheless, it is important to highlight that even in the context of a downstream inflammatory response, immune dysregulation may still impact AD pathological cascades and clinical manifestation of the disease.

It is now clear that immunologically relevant molecules circulating in the blood can have a direct and lasting impact on brain function. This has been demonstrated using animal models, perhaps most directly by heterochronic parabiosis in which young mice are transfused with plasma from aged mice, resulting in reactive microglial response and reduced hippocampal neurogenesis in young rodents [27]. Additionally, peripheral administration of lipopolysaccharide (LPS), an endotoxin found on the outer membrane of gram-negative bacteria, has been used at low doses to recapitulate the effects of mild infection and peripheral inflammation. Using this method, several studies have demonstrated that low-grade inflammatory insults can exacerbate the progression of AD pathology [28, 29].

### Acute inflammatory insults in Alzheimer’s disease

The aging process itself is characterized by changes in both innate and adaptive immune responses often referred to as immunosenescence. This process affects both myeloid and lymphoid cells, where it is characterized by an upregulation in pro-inflammatory signaling in the context of immune activation and a reduction in naïve lymphocytes, antibody effectiveness, and phagocytic capacity, the latter of which allows for accumulation of noxious proteins peripherally and within the CNS in a manner which further propagates immune activation [30–32]. Senescent immune cells have been causally implicated in nonlymphoid tissue (including brain) aging, and are more susceptible, compared to healthy immune cells, to producing an amplified and protracted inflammatory response in the context of an acute inflammatory insult [33–35].

**Fig. 1:**
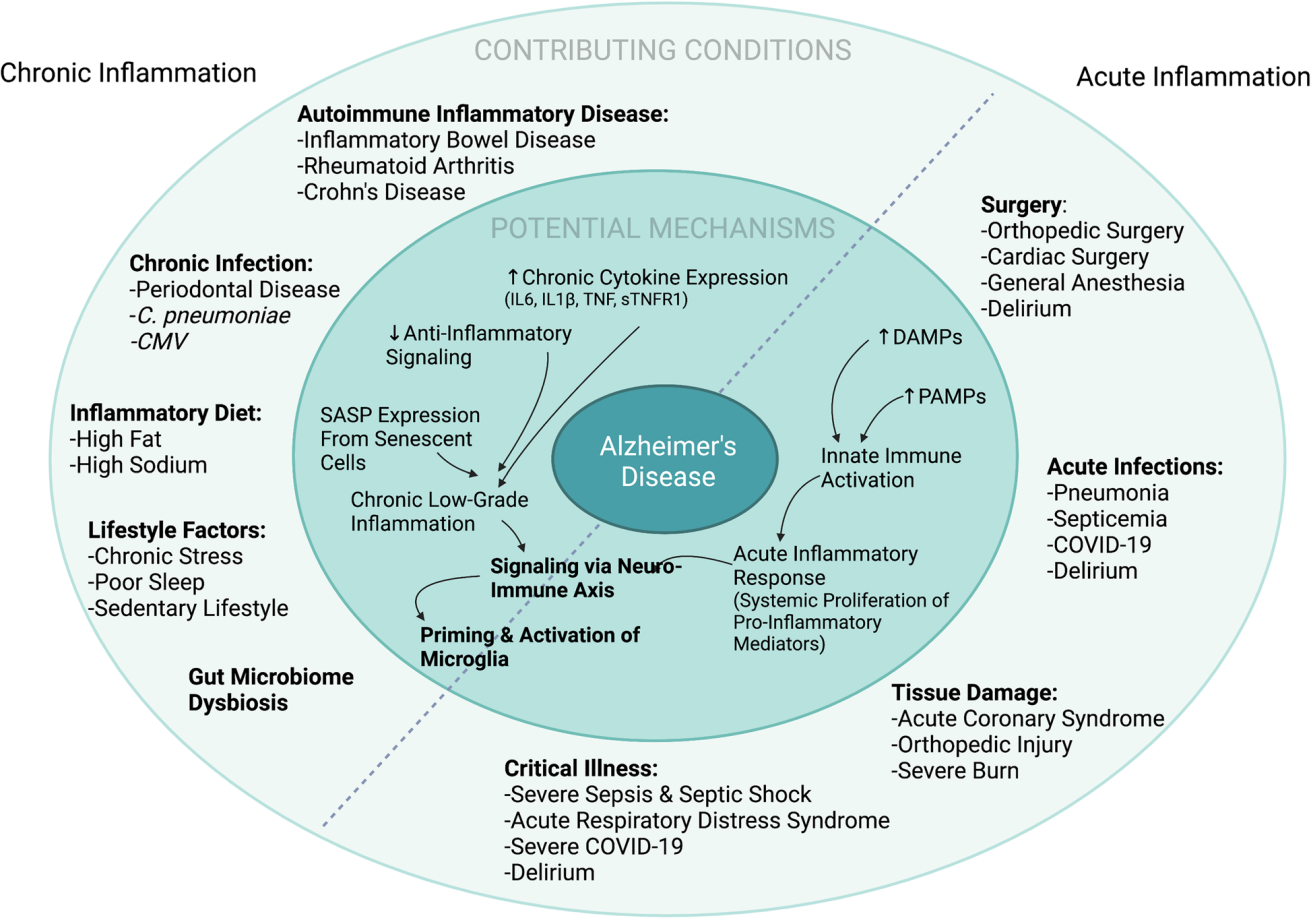
Contributing conditions and potential mechanisms underlying the relationship between inflammation and Alzheimer’s disease. The outer circle specifies several acute and chronic conditions that have been hypothesized to influence risk for Alzheimer’s disease and related dementias. The inner circle specifies potential biological mechanisms by which acute and chronic inflammatory conditions may influence target cells within the brain, specifically microglia. The biological processes initiated by acute and chronic inflammatory conditions are hypothesized to converge on common signaling pathways that can in turn prime and activate microglia via the neuro-immune axis

The immune response to acute inflammatory events differs from chronic low-grade inflammation across multiple dimensions, including triggers, timing (duration), and magnitude. Acute inflammatory stimuli typically come in the form of pathogen exposure or tissue damage, both of which can range markedly in severity. Chronic inflammation, on the other hand, typically represents a response to chronic perturbations to homeostasis (Fig. 1). The immune response to acute insults is tightly regulated and temporally limited in younger adults, whereas in older adults the response to acute insults may be more protracted [18, 36, 37] and thus more likely to initiate or catalyze ongoing degenerative processes [38]. The time-limited upregulation of inflammatory cytokines that occurs following an acute inflammatory insult is many fold times higher in magnitude than that observed in the context of chronic inflammation [39, 40]. Moreover, temporal specificity has been noted for some molecular mediators, such as IL-8, IL-16, and G-CSF, which appear at elevated levels in the context of acute, but not chronic, inflammation [41]. The distinction between acute and chronic inflammatory responses is not always clear, however. For example, chronic inflammatory responses can often follow when an acute inflammatory response has been unsuccessful [42].

## Infection as a risk factor for Alzheimer’s disease

Historical interest in a possible link between infection and *distal* neurological outcomes has been present for more than a century, although establishing direct mechanistic connections between the two in human populations has been challenging. The association between acute infections and risk of dementia has been reported in numerous epidemiological studies[43–45] (Table 1 and Supplementary Table 1). These studies have leveraged large databases of older adults to assess the relationship between acute infection and AD/dementia risk. For example, one study found that compared to patients *without* a diagnosis of AD dementia, patients with AD were more likely to have been evaluated for a urinary tract infection during the 36 months prior to receiving their dementia diagnosis [46]. Appraising the relationship between a broader range of infections and dementia, a retrospective cohort study of US veterans (age > 55) found a link between non-CNS bacterial infection and risk for future dementia, with independent effects of bacteremia, osteomyelitis, pneumonia, UTIs, cellulitis, and sepsis on dementia risk [47]. In contrast, studies do not show a consistent relationship between minor infections (e.g., influenza) and AD risk in older adults (age > 64) [48].

**Table 1:** Studies examining the association between multiple infection categories and Alzheimer’s disease, vascular dementia, all-cause dementia

Author, Year	Cohort/Patient Registry or Country (Total sample size)	Infection Type	Infection Diagnostic Method	Outcome (Follow-Up Years^*^)	Results
Sipila et al., 2021 [49]	Finnish multicohort (*n* = 260,490) UK Biobank (*n* = 485,708)	Bacterial Viral CNS (present/absent; frequency)	HDR	ACD, AD, VaD (primary cohort, 19; replication cohort, 4)	Any infection, infection frequency, viral, bacterial, CNS: ^↑^ACD, ^↑^AD, ^↑^VaD
Douros et al., 2021 [50]	CPRD (*n* = 4,262,092)	HSV1/2 *Borrelia burgdorferi* *Porphyromonas gingivalis* *Escherichia coli* *Helicobacter pylori* *Chlamydophila pneumonia* *Candida albicans* (present/absent; frequency)	EHR	AD (30)	Any infection, *H. pylori*: ^↑^AD HSV1/2: –AD *B. burgdorferi*: –AD *P. gingivalis*: –AD *E. coli*: –AD *C. pneumonia*: –AD *C. albicans*: –AD Frequency: –AD
Bohn et al., 2023 [51]	ARIC study (*n* = 15,688)	Any Infection, respiratory, urinary tract, skin, blood/circulatory system, intestinal (present/absent)	HDR	ACD (32)	Any Infection, respiratory, urinary tract, skin, blood/circulatory system: ^↑^ACD Intestinal: –ACD
Levine et al., 2023 [52]	FinnGenn (*n* = 344,189) UK Biobank (*n* = 106,066)	73 different viruses (present/absent)	HER	ACD, AD, VaD (not reported)	Influenza: ^↑^ACD, ^↑^VaD Viral pneumonia, warts, encephalitis, ‘other’: ^↑^ACD Influenza + pneumonia: ^↑^ACD, ^↑^AD, ^↑^VaD Viral intestinal: ^↑^ACD, ^↑^AD, ^↑^VaD Meningitis, viral encephalitis: ^↑^AD VZV: ^↑^VaD
Mawanda et al., 2016 [47]	Veterans Health Administration (*n* = 417,172)	Pneumonia Septicemia Bacteremia Urinary tract infections Cellulitis Septic arthritis Osteomyelitis (present/absent)	HER	ACD (12)	Any infection: ^↑^ACD Pneumonia: ^↑^ACD Septicemia: ^↑^ACD Bacteremia: ^↑^ACD Urinary tract infection: ^↑^ACD Cellulitis: ^↑^ACD Septic arthritis: –ACD Osteomyelitis: ^↑^ACD
Muzambi et al., 2021 [53]	CPRD (*n* = 989,800)	Sepsis Pneumonia Other lower respiratory tract infections Urinary tract infections Skin/soft tissue infections (present/absent; frequency)	HER	ACD AD VaD Other dem. (15)	Any Infection: ^↑^ACD, ^↑^AD, ^↑^VaD, ^↑^Other dem Frequency: ^↑^ACD Sepsis: ^↑^ACD, ^↑^VaD, ^↑^Other dem Pneumonia: ^↑^ACD, ^↑^AD, ^↑^VaD, ^↑^Other dem Other lower respiratory tract infections: ^↑^ACD, ^↑^VaD, ^↑^Other dem Urinary tract infections: ^↑^ACD, ^↑^AD, ^↑^VaD, ^↑^Other dem Skin/soft tissue infections: ^↑^ACD, ^↑^AD, ^↑^VaD, ^↑^Other dem
Sun et al., 2022 [54]	NPR (*n* = 1,751,646)	Bacterial Viral CNS Respiratory Gastrointestinal Skin Urinary tract infections (present/absent)	EHR + HDR	AD (46)	Any infection: ^↑^AD Bacterial: ^↑^AD Viral: ^↑^AD CNS: ^↑^AD Respiratory: ^↑^AD Gastrointestinal: ^↑^AD Skin: ^↑^AD Urinary tract infections: ^↑^AD
Mekli, et al., 2022 [55]	UK Biobank (*n* = 9,431)	17 viruses (present/absent)	Serum IgGs	ACD (9)	HSV1: ^↑^ACD HSV1 + HHV6 + HHV7 + VZV: ^↑^ACD HSV2, EBV, VZV, CMV, HHV6, HHV7, HHV8, HBV, HTLV, BKV, JCV, HPV, MCV: –ACD
Dunn et al., 2005 [43]	GPRD (*n* = 19,328)	Chest Genitourinary Skin Other (frequency)	EHR	ACD (10)	Frequency: ^↑^ACD
Strandberg et al., 2003 [56]	Finland (*n* = 383)	HSV1 HSV2 CMV *Chlamydia pneumoniae* *Mycoplasma pneumoniae* (frequency)	Serum IgGs	MMSE (1)	Viral frequency: ↓MMSE Bacterial frequency: –MMSE
Wright et al., 2015 [57]	NOMAS (*n* = 419)	HSV1 HSV2 CMV *Chlamydia pneumoniae* *Helicobacter pylori* (frequency)	Serum IgGs	Language, Memory, Executive function Processing speed (14)	Frequency: –Language, Frequency: ↓Memory Frequency: –Executive function Frequency: –Processing speed

Abbreviations: *ACD* all-cause dementia, *AD* Alzheimer’s disease, *ARIC* Atherosclerosis Risk in Communities study, *BKV* human polyomavirus BKV, *CMV* cytomegalovirus, *CNS* central nerouvs system, *CPRD* Clinical Practice Research Datalink, *EBV* Epstein–Barr virus, *EHR* electronic health records, *HBV* hepatitis B virus, *HDR* hospital discharge records, *HHV* human herpes virus, *HPV* human papillomavirus, *HSV* herpes simplex virus, *HTLV* human T-cell lymphotropic virus, *IgG* immunoglobulin G, *JCV* human polyomavirus JCV, *MCV* Merkel cell polyomavirus, *MMSE* Mini-Mental State Examination, *NOMAS* Northern Manhattan Study, *VaD* vascular dementia, *VZV* varicella zoster virus

^↑^infection associated with increased dementia risk/cognitive performance

^↓^infection associated with decreased dementia risk/cognitive performance; – null association

^*^Maximum follow-up time/observational window

The prior studies raise several questions regarding the role of infections in the development of AD, including whether there is differential risk for AD in late life based on the severity or type of infection. Additionally, given that the AD pathology is known to occur at least 1 to 2 decades before symptom onset, it remains unclear whether acute infections occurring proximal vs distal to dementia onset play a mechanistic role in AD development. Sipilä and colleagues (2021) directly addressed this question by evaluating in a multi-cohort study the long-term relationship between hospital-treated viral and bacterial infections and risk for AD and non-AD dementias [49]. The authors demonstrated a) that hospitalization for any infection was associated with increased risk of dementia, with little specificity for type or severity of infection; b) a dose–response relationship between the number of discrete hospital-treated infections and greater risk of dementia; and c) stronger associations between infections and vascular dementia than between infections and AD dementia. Importantly, the primary findings remained robust when analyses were restricted to infections which occurred more than 10 years prior to dementia diagnosis, however the effect sizes were attenuated. Similar findings were also recently reported in large, retrospective studies which demonstrated that common infections (e.g., sepsis, pneumonia, influenza, and skin and soft tissue infections) increased risk for dementia over a multi-decade follow-up period [51–53, 58]. Acute, immune-related health events have also been associated with exacerbated clinical presentation of AD as well as more rapid cognitive decline in community-dwelling older adults. A seminal study demonstrated that recent systemic inflammatory events (e.g., non-CNS infection) in older adults were associated with elevated TNF-ɑ levels and a two-fold increase in the rate of cognitive decline over a six-month period [38]. Remarkably, individuals with elevated TNF-ɑ at baseline who subsequently experienced a systemic inflammatory event tended to show an even greater rate of cognitive decline. Collectively, these results suggest that peripheral infections, even infections that occur a decade or more before dementia onset, may increase dementia risk, presumably via systemic inflammatory mechanisms and vascular conduits. More studies are needed, however, to determine whether there is a critical period of the lifespan (i.e., midlife vs late life) or a critical period of AD pathogenesis during which exposure to an acute infection yields more pernicious effects on cognitive outcomes.

The COVID-19 pandemic has brought the short- and long-term effects of infection to the forefront of research and public health. Like other acute infections, COVID-19 is likely to be associated with residual decrements in cognition. The rapidly evolving literature includes reports which suggest that 30–40% of COVID-19 patients experience memory and concentration issues within the first 100 days after hospitalization [59]. It is not yet confirmed whether these symptoms are chronic or worsen/accelerate existing neurodegenerative processes [60], but there is already evidence that SARS-CoV-2 infection is associated with near-term reductions in cortical thickness, brain volume, cognition [61]. There does appear to be overlap in disease biology between COVID-19 and AD, as demonstrated by network and transcriptomic analyses which show overlap in neuroinflammatory and vascular injury pathways [62]. The interplay of SARS-CoV-2 with host immune processes could be a particularly important mechanism. While the acute phase of SARS-CoV-2 infection can be marked by viral evasion of the innate immune system, the post-acute phase is characterized by exaggerated myelopoiesis and prolonged expression of type I and type III interferons (INFs) and inflammatory cytokines [63]. Higher circulating INF-**γ**, INF- β, and cytokines levels in the months following COVID-19 infection are in turn associated with post-acute sequelae of SARS-CoV-2 (PASC) [64]. However, whether these mechanisms actually increase risk for AD remains to be seen. Epidemiological studies suggest that carriers of *APOE*ε4, the major AD risk allele, are more likely to test positive for and die from COVID-19, a phenomenon which may be due in part to reduced antiviral defense among *APOE*ε4 carriers [65]. Global consortia have established large cohorts which will be critical for understanding the long-term neurological consequences of COVID-19 on the central nervous system [66].

## Critical illness as a risk factor for Alzheimer’s disease

Critical illness represents another common acute inflammatory insult associated with both short- and long-term cognitive impairment. Critical illness includes severe sepsis, septic shock, acute respiratory distress, and cardiogenic shock, conditions which necessitate life-saving interventions administered within the intensive care unit (ICU). Although there is a great deal of heterogeneity in the physiological response to critical illness, common to this set of conditions is a severe and often systemic immune response marked by acute inflammation, and in some cases, secondary immunosuppression [67, 68]. While both inflammation and immunosuppression can be deadly, the inflammatory response is believed to be a primary driver of acute brain dysfunction or delirium [69]. Cognitive dysfunction among critically ill patients has been shown to persist beyond the ICU, with several large prospective studies having demonstrated a link between critical illness and long-term cognitive impairment [70–72]. A seminal study of 821 adults admitted to the ICU who experienced septic/cardiogenic shock or respiratory failure found that 34% and 24% of patients had a global cognitive score more than 1.5 and 2 standard deviations below the population mean, respectively, at 12 months after discharge [71]. For critical illness survivors, delirium and delirium duration have been identified as among the most consistent predictors of post-discharge cognitive impairment, suggesting that (1) a common pathophysiology may underlie delirium and longer-term cognitive deficits and/or (2) delirium itself may be a cause of long-term cognitive deficits [71–73].

While critical illness has been associated with cognitive impairment, at least for a subset of patients, few studies have directly examined the association between critical illness and AD or dementia risk. A study of Medicare beneficiaries found a de novo diagnosis of dementia was made in 18% of participants who received intensive care over the three-year follow-up period. In this study, severe sepsis, infection, and acute dialysis were among the factors associated with dementia [74]. Supporting these findings, a retrospective study of the Taiwan Longitudinal Health Insurance Database found that sepsis was associated with dementia diagnosis over a five-year period [75]. Given the short period of time between the critical illness and the diagnosis of dementia in these and other studies [76, 77], it is difficult to draw conclusions about the directionality of the association. One large European study with a 30-year follow-up period found that a history of critical illness hospitalization was associated with increased risk of subsequent AD dementia and all-cause dementia, supporting the hypothesis that critical illness is indeed a dementia risk factor [78].

Numerous mechanisms have been proposed to link critical illness with acute and long-term cognitive deficits. Infections, including ventilator-associated pneumonia and bloodstream infections, are common in the critical care setting, especially among older patients. The link between critical illness, infection, and Alzheimer’s disease has been proposed to be mediated through endotoxin exposure. Endotoxins, a type of LPS found on the outer membrane of gram-negative bacteria, are elevated in the bloodstream in the context of peripheral infections and acute illness (e.g., septic shock) [79]. Endotoxins interact with myeloid cells via pattern recognition receptors, including TLR2, TLR4, TREM2, and scavenger receptors to increase peripheral cytokine expression and activate brain endothelial cells via NF-kB [80]. Through peripheral-to-central immune signaling pathways (described below) endotoxins can activate microglia, increase brain cytokine levels, and drive Aß aggregation and hyperphosphorylation of tau [81]. Endotoxins may also promote neuro-immune activation directly by gaining entry to the brain and activating microglia, for example, via TLR2 signaling [82]. Although it is not yet clear whether critical illness itself – independent of infection or microbial exposure – represents a risk factor for cognitive decline and AD, evidence suggests that non-infectious factors common in critical illness, such as hypoxemia in the setting of respiratory distress, vascular injury and blood–brain barrier (BBB) dysfunction in the setting of cardiogenic shock, and exposure to sedatives and anesthetic agents, may also contribute to cognitive decline [83–85]. Importantly, risk factors and biological drivers of near-term cognitive impairment following critical illness are likely distinct from those of long-term cognitive decline and dementia risk. Such distinctions will need to be addressed in future observational and mechanistic studies.

Neuroimaging and neurophysiological studies offer additional clues about the neurological underpinnings of cognitive decline following critical illness. Compared to non-hospitalized controls, lower hippocampal volumes as well as greater low-frequency EEG activity (a non-specific indicator of brain dysfunction) was found in sepsis survivors 6–24 months after discharge from the hospital [86, 87]. Similarly, reduced brain volume in temporal-parietal brain regions vulnerable to AD-related atrophy was found in older adults who experienced one or more critical illnesses or hospitalized infections in the preceding two decades [88]. These findings support the notion that critical illness may heighten one’s risk for dementia through neurodegenerative and physiological brain changes.

## Surgery and aseptic insults as risk factors for Alzheimer’s disease

Surgery, though typically considered to be a restorative medical procedure, is often characterized by some degree of tissue damage. Whether it results from a planned and well-controlled operation or from a physical trauma, such as a hip fracture or severe burn, tissue damage causes systemic expression of inflammatory proteins. Although studies designed to examine the cognitive effects of surgery have yielded mixed results, post-operative cognitive decline (POCD) in the weeks and months following surgery has been estimated to be fairly prevalent, occurring in approximately 30% of individuals [89]. However, in the post-operative timespan ranging from one month to one year, evidence for POCD is less consistent. Many studies, especially those which employ control groups, show little to no evidence of elevated rate of POCD within this period [89]. A meta-analysis of 17 studies found no evidence for cognitive decline in the 3 to 6 months following total joint arthroplasty (hip and knee replacement) [90]. However, a study examining POCD at one-year follow-up in patients who underwent elective surgery found severe POCD in 11% of surgery patients, compared to 4% of non-surgical control participants [91]. Inconsistent findings may be due, at least in part, to differences in type of surgery and other pre- and peri-operative variables, such as patient-specific health, depth of anesthesia, and the occurrence of postoperative delirium.

In light of evidence for delayed neurocognitive recovery, there has been considerable interest in understanding the long-term effects of surgery on cognition and subsequent dementia risk [92]. It has been proposed that individuals who experience POCD may be at risk for experiencing permanent or delayed cognitive decrements following surgery. Within the Rochester Epidemiology Project and the Mayo Clinic Study of Aging, surgery/general anesthesia within the past 20 years was associated with a 30% increase in risk for MCI [93]. This finding has been replicated for dementia using a large Taiwanese medical claims database [94]. A Danish study of middle-age and elderly twins found very modest decrements in cognition associated with surgery within the past 18 years [95]. A similar study from the Swedish Twin Registry found an approximate 30% increase in AD and all-cause dementia risk associated with surgical hospitalization in a registry-based sample [78]. However, this relationship was attenuated in a within-monozygotic-twin-pair comparison, suggesting that a shared genetic predisposition for surgical hospitalization and dementia may account, at least in part, for the associations observed between surgery and dementia.

Supporting the association between surgery and neurocognitive changes, fluid biomarker studies using pre- and post-operative plasma and CSF measurements have found evidence of post-operative increase in neuronal injury proteins (total tau, NSE, and NfL) within the first 48 h after surgery [96, 97]. Neuroimaging has shed additional light on this issue by relating surgery to metrics of brain health measured many years later [98, 99]. For example, a recent study found that exposure to surgery with general anesthesia in the preceding 20 years was associated with reduced cortical thickness in regions vulnerable to atrophy in AD, particularly the entorhinal cortex [100]. Currently, evidence for an association between surgery and AD neuropathology is mixed. One study of 10 patients measured CSF before and 24 h after open heart surgery and found acute increases in Aβ_1–42_. However, the relative increases in CSF TNF-ɑ, IL-6, and IL-8 were more marked, suggesting that increased neuroinflammatory signaling may be among the most prominent changes, at least in the short term [101]. Supporting these results, a small study of patients who had surgery for an idiopathic nasal correction found no postoperative increases in CSF Aβ_1–42_, but did find increases in CSF markers of inflammation and neuronal/glial injury 24 h after surgery [102]. Similarly, changes in CSF and plasma Aβ_1–42_ were found to occur in the 48 h after orthopedic surgery; however, measures of post-surgical neuroinflammation, but not post-surgical Aβ, were associated with cognitive status months after surgery [96, 103]. Two recent cohort studies have failed to find definitive support for a long-term association between surgery and brain Aβ levels defined using PET imaging [104–106]. Together, these findings suggest that the potential effect of surgery on cognitive decline and dementia risk may occur through neuro-immune pathways that are independent of Alzheimer’s pathology such as amyloid.

Multiple lines of translational and clinical research support the hypothesis that nonpathogenic (sterile) inflammatory triggers, such as surgery, can have short- and long-term effects on neurobiology, cognition, and dementia risk. The tissue damage resulting from invasive surgical procedures causes a systemic expression of endogenous molecules called damage associated molecular patterns (DAMPs). DAMPs are released in response to cellular stress and injury and include molecules such as high-mobility group-box chromosomal protein 1 (HMGB1), heat shock proteins, S100 proteins, IL-1a, and IL-33 [107]. DAMPs interact with pattern recognition receptors on myeloid cells in a process that activates the innate immune system. This aseptic inflammatory response is typically short-lived in healthy young adults, but it can be perpetuated in older individuals. Studies have demonstrated significant elevations in circulating inflammatory proteins, such as TNF-ɑ, in blood as early as 30 min after surgery. This is followed by elevations in other cytokines (including IL-1β and IL-6) and chemokines in blood occurring in the hours and days following surgery [103, 108–110]. This systemic immune activation, through one or more of the peripheral-to-central mechanisms of immune crosstalk causes an upregulation of inflammatory mediators within the brain (e.g., IL1ß, HMGB, complement C3) resulting in further activation of microglial and reactive astrocytes [110–112]. Through processes such as cytokine-mediated inhibition of synaptic plasticity [113], astrocyte-mediated neurotoxicity [114], and complement-mediated impairments in synaptic pruning [115], neuroinflammation may contribute to neurodegenerative processes following surgery.

Sterile inflammation can result from DAMP signaling of multiple families of pattern recognition receptors, including TLRs, NOD-like receptors (NLRs), AIM2-like receptors, RIG-I-like receptors (RLRs), and C-type lectin receptors (CLRs), many of which also recognize PAMPs that signal pathogen exposure [116, 117]. Overall, the inflammatory signaling pathways activated in the context of tissue damage are similar to those activated in the context of infection [42, 116]. Despite many similarities, tissue injury is more likely than pathogen exposure to result in an inflammatory remodeling process capable of changing the tissue composition and architecture (e.g., fibrosis) [42, 118]. Notably, pathogen exposure may also cause secondary tissue damage in a manner which promotes further immune activation, blurring the distinction between tissue-injury and pathogen mediated inflammation [116]. In addition to the immune-mediated pathways described above, there are several other mechanisms through which surgery may contribute to subsequent cognitive decline, including changes to cerebral perfusion or autoregulation, reduced arterial oxygenation, or neurochemical changes associated with anesthesia exposure [119, 120].

## From peripheral inflammatory insults to neuroinflammation: Mechanisms and pathways

Though human research has been informative in this area, much of the understanding of how peripheral immune challenges interact with neurobiological processes to influence brain health has come from translational research. Models of infection, critical illness, and injury have allowed investigators to identify the mechanisms of peripheral-to-central crosstalk and the peripherally derived cellular and molecular mediators affecting target cells within the CNS.

Acute peripheral bacterial infection has been associated with sustained elevations in hippocampal IL-1β levels [121], whereas peripheral infection with influenza has been found to induce neuroinflammatory pathways and impair hippocampal plasticity [122]. One recent study demonstrated an amplified cytokine response in aged mice relative to younger mice in response to a viral RNA analogue (poly I:C) [123]. This amplified inflammatory response occurred in both peripheral circulation and the hippocampus, the latter of which was associated with working memory deficits. Of note, neuroinflammation and cognitive deficits resulting from infection may be caused, at least in part, by the generation of NO by NOS2, as NOS2 knockout animals were protected from sustained microglial activation, expression of proinflammatory factors in brain, and behavioral deficits [124]. While these studies suggest that acute peripheral infections may cause short-term neuroimmune changes and alterations to brain morphology and cognitive function, compelling data also suggest that acute infections may directly affect the development and progression of AD. Recent studies have, for example, demonstrated that exposure to LPS compromises microglial Aβ phagocytosis in APP/PS1 mice, leading to a persisting accumulation of Aβ deposits in the neocortex [125]. Similar results have been derived from animal models of sepsis and other forms of critical illness [126, 127]. In the context of aseptic inflammation, rodent models have also demonstrated evidence of microgliosis and increased transcription of inflammatory genes in the brain shortly after surgery [108, 110]. Prophylactic inhibition of peripheral TNF-ɑ before surgery has been associated with reduced post-surgical inflammation, microgliosis, and cognitive impairment, highlighting the contribution of peripheral TNF signaling to postoperative brain changes [110].

**Fig. 2:**
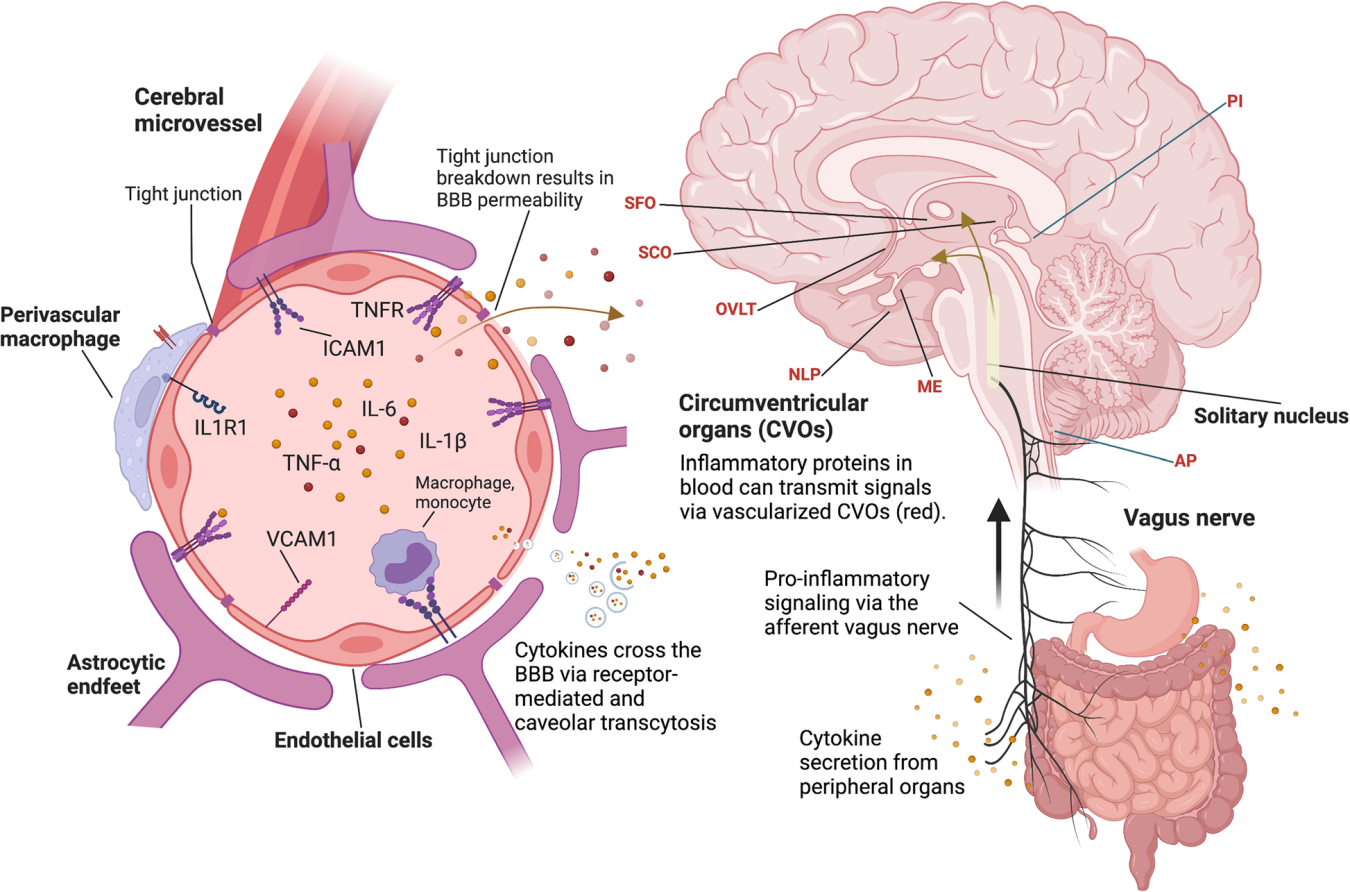
Peripheral inflammation affects brain function via the neuro-immune axis. Levels of inflammatory proteins increase in the blood after an inflammatory insult. Circulating inflammatory proteins can activate endothelial cells, which then upregulate cellular adhesion molecules, such as ICAM1 and VCAM1. Cellular adhesion molecules tether myeloid cells, leading to further endothelial activation and vascular inflammation. As part of this process endothelial cells activate pro-inflammatory transcription factors, causing an increased expression of pro-inflammatory factors in the brain. Circulating inflammatory proteins in blood (e.g., TNF-ɑ) can cause breakdown in blood–brain barrier tight junctions, allowing for increased flow of blood proteins into the brain parenchyma and CSF. Inflammatory proteins in blood can also be transported into the brain via receptor-mediated transcytosis, and with increasing age, non-specific caveolar transcytosis. Outside the cerebral microvessel, activation of perivascular macrophages can further enhance blood–brain barrier permeability. Comprised of fenestrated capillaries, circumventricular organs (listed in red text) are gaps within the blood–brain barrier that allow for direct communication between molecules circulating in the blood, including cytokines, and target cells within the brain. In the context of peripheral inflammation, the vagus nerve sends inflammatory signals from the gut, liver, lungs, and other organs to the brain. The solitary nucleus relays these peripherally derived inflammatory signals to the hypothalamus, thalamus, and other brain regions in a manner which can promote glial expression of inflammatory proteins and receptors. *Abbreviations*: AP, area postrema; ICAM1, intercellular adhesion molecule 1; IL-1β, interleukin 1 beta; IL1R1, interleukin 1 receptor type 1; IL-6, interleukin 6; ME, median eminence; NLP, neural lobe of the pituitary gland; OVLT, organum vasculosum of the lamina terminalis; PI, pineal gland; SCO, subcommissural organ; SFO, subfornical organ; TNF-ɑ, tumor necrosis factor alpha; TNFR, tumor necrosis factor receptor; VCAM1, vascular cell adhesion molecule 1

There are multiple routes through which peripherally secreted pro-inflammatory factors, such as TNF-ɑ, may influence target cells within the brain. The BBB, which acts as a critical interface for maintaining brain homeostasis, represents one of the most important and widely studied conduits for peripheral-to-central immune crosstalk. This structure is part of the neurovascular unit (NVU) and is comprised of a continuous endothelial cell (EC) layer with tight cell-to-cell contacts, sheathed by an endothelial and parenchymal basement membrane, pericytes, and perivascular astrocytes [128]. The BBB has a key role in protecting the CNS from systemic factors including pathogens, inflammatory molecules, and immune cells. BBB dysfunction is often identified by the loss of tight junctions (TJs), alterations in transport properties, and changes in adhesion molecule expression (Fig. 2). BBB dysfunction can lead to an influx of systemic factors (e.g., fibrinogen), inflammatory mediators, and immune cells into the brain parenchyma [129–131]. [132]. Acute infective and aseptic insults can transiently alter BBB properties, and in doing so may precipitate or perpetuate CNS vulnerabilities. For example, orthopedic surgery in mouse model has been associated with a loss of TJ expression (i.e., claudin-5) in the hippocampus [133]. Similar rodent studies have demonstrated that exposure to other surgical procedures results in alterations in BBB properties and subsequent neuroinflammation [134, 135]. BBB dysfunction has also been implicated in AD pathogenesis, as impaired BBB was found to trigger the acute deposition of Aβ in the brains of CVN-AD mice (APPSwDI/NOS2^−/−^) following orthopedic surgery [136].

As illustrated in Fig. 2, there are several other conduits through which peripheral inflammation may lead to neurobiological changes. Neural pathways include vagal afferents to the brainstem, in which peripheral inflammatory insults and/or systemic inflammation stimulate vagus nerve pathways that signal to the hypothalamus via the solitary tract. Humoral pathways may involve activation of or direct transport across specific barrier interfaces, including the choroid plexus (i.e., blood-CSF barrier) as well as other circumventricular organs (CVOs; e.g., area postrema) [137, 138]. The choroid plexus has been of particular interest to the immunity-AD field given its role not only in CSF production but also broad immune surveillance and immunoregulation [139–141]. For example, a recent study demonstrated that peripheral inflammation induced immune cell infiltration into the brain via choroid plexus in wild type mice and in an APP knock-in (*App*^*NL−G−F*^*)* mouse model. In the context of peripheral inflammation, the choroid plexus upregulated expression of integrin ligands and chemokines (ICAM1, CCL2, and CXCL10), a process which can induce leukocyte trafficking into the brain [141].

The meningeal lymphatic system has also been proposed to play a central role in connecting the peripheral immune response with neuroinflammation. The meningeal immune compartment, unlike the brain itself, is populated with a diverse set of immune cells under normal physiological conditions [142, 143]. The meninges sit directly above the brain and because there is no BBB protecting the meninges, can be directly accessed by peripheral immune cells via the lymphatic system. The activation of meningeal macrophage and innate lymphoid cells has been shown to cause T cells, monocytes, and neutrophils to infiltrate the parenchyma in a manner which promotes CNS inflammation and tissue damage [144–146]. Given the close connection between the peripheral immune system and meningeal immune compartment, peripheral inflammatory events could transiently, or perhaps even chronically, alter immune function within the brain via a process of meningeal remodeling referred to as lymphangiogenesis [147–149]. For example, one study found that viral infection of the meninges resulted in changes to the phenotype and function of meningeal macrophage, altering their response to subsequent immune challenges [147]. Whether changes to the meningeal immune compartment occur following acute infection, major surgery, or other peripheral inflammatory insults is not yet known.

In contrast to the lymphatic system, which may allow peripheral immune processes to influence the CNS, the glymphatic system is considered to function primarily as a mechanism for CNS clearance [150] and therefore may allow neuroimmune processes to influence peripheral immunity via the transport of molecules from the parenchyma into the cervical lymphatic drainage [151]. One study has demonstrated that a peripheral immune challenge (endotoxin exposure) caused a reduction glymphatic drainage (i.e., perivascular flow of CSF) at three hours after the exposure; however, the mechanisms linking peripheral immune activation with glymphatic changes remain unknown [152].

## Next steps: a research roadmap

**Fig. 3:**
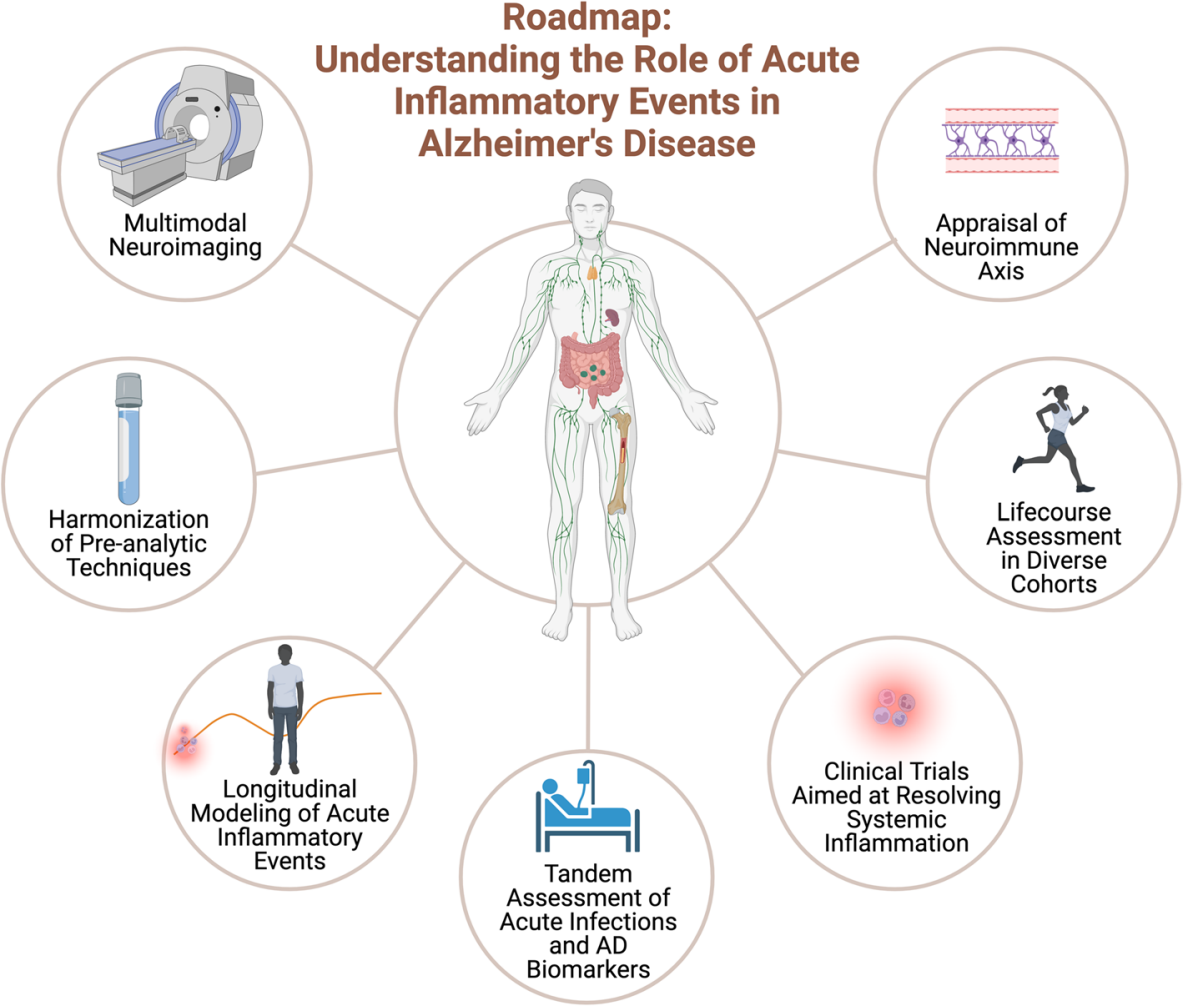
Roadmap: Acute inflammatory events and Alzheimer’s disease To overcome prior barriers to progress in the immunity-AD field, longitudinal, multimodal appraisal of acute inflammatory events in diverse cohorts is needed. A proposed roadmap of studies needed to address gaps in the literature is depicted, focusing on biological, neuroimaging, and environmental assessments of the relationship between acute inflammatory exposures and long-term outcomes, as well as the development of interventional trials aimed at resolving systemic inflammation

In the section that follows, we provide a roadmap for future research efforts directed at (1) understanding the role that peripheral inflammatory insults may play in AD pathogenesis, and (2) developing interventions to limit the deleterious effect these inflammatory stressors may have on brain health (Fig. 3).

### Longitudinal studies to disentangle the temporal dynamics of acute infection in AD

Given that acute inflammatory insults occur throughout the lifespan and are typically poorly characterized in cohort studies, delineating the temporal relationship between such inflammatory insults and AD development has been challenging. Furthermore, it is unclear how the type, duration, or number of systemic inflammatory exposures catalyzes the AD pathological cascade, and whether such exposures indeed modify the disease course and are additive in nature.

As noted previously, recent studies [153] indicate that infections resulting in hospital treatment are related to future dementia risk, even after modeling specific exposure periods to minimize reverse causality and ascertainment biases. Results from these studies highlight the possibility that the mechanism by which infections impact AD pathogenesis is via general inflammation and vascular pathways rather than a specific microbe/pathogen. However, several questions remain regarding pathology, timing, and dose effects. For example, to determine whether exposure to acute inflammatory insults is pathogenic at specific stages of the Alzheimer’s disease process, careful prospective evaluation of individuals stratified using AD biomarker frameworks (e.g., A/T/N) will be important. Ascertaining longitudinal AD biomarkers in the context of acute inflammatory insult exposures should also provide insights into short- and long-term consequences of these immune challenges across critical life stages (i.e., midlife, late life) and pathological states (i.e., AD continuum biomarker profiles). Additionally, longitudinal studies that evaluate systemic inflammation and components of the neuro-immune axis (e.g., BBB integrity) following an acute inflammatory event will provide further insight into the pathways that connect peripheral inflammatory insults to changes to the CNS milieu. That said, focusing solely on peripheral inflammatory biomarkers will likely be insufficient to understand the complex sequence of biological events that follows the clinical resolution of an acute infection. Leveraging genetic data, multi-omic profiling, and neuroimaging in tandem with acute inflammatory event history and AD-related fluid biomarkers over time will provide much needed insights into timing effects and possible interactions between putative AD risk factors.

Extending this further, burgeoning evidence points to changes in the adaptive immune system in aging adults and adults with AD, with recent data showing that a portion of clonally expanded CD8 + T-cells in AD patients may be specifically reactive to the Epstein-Bar virus [154]; although outside the scope of this Roadmap paper, it will be important to clarify how acute inflammatory insults relate to chronic or latent infections, how acute infectious and non-infectious inflammatory insults impact the adaptive immune response, and whether chronic infections alter the acute inflammatory response in older adults over time [155]. The presence of co-pathology in aged brains adds increasing complexity to this framework, as late life acute exposures occur in a CNS milieu defined by the presence of multiple pathologies and heterogeneous immune states. Disentangling chronic inflammatory and/or adaptive immune responses to both AD and non-AD pathology from the effects of acute insults will be a challenging, but critical goal for the field. Ultimately, these efforts will require interdisciplinary collaborations between AD researchers and those involved in the care and study of systemic disease and physiology.

Finally, another understudied area of research on acute inflammatory insults is how sex-based differences in immune response might serve as potential contributors to AD risk. A large body of evidence suggests that while females generally display stronger innate and adaptive immune responses, clear some infections more rapidly, and show greater antibody response to vaccines, they are also at increased risk for autoimmune diseases compared to males [156, 157]. Sex differences in immune responses have also been shown to change across the lifespan [156]. Considering that immune dysregulation is a core feature of AD and females are at greater risk for AD relative to males, studies that examine how sex differences might impact immune-mediated contributions to AD pathogenesis and progression are clearly needed. Taking this one step further, longitudinal studies will need to examine the role of sex-based differences in immune response to *acute* inflammatory insults and map results onto AD biomarker status and future risk of AD in order to better elucidate the role of sex effects on both immediate and downstream pathological cascades.

### Scalable neuro-immune axis biomarkers: Measuring BBB integrity

Efforts to identify and understand the pathways through which peripheral inflammation may exert effects on the aging human brain have been hampered due to a limited capacity to assess peripheral-central immune crosstalk and the integrity of conduits, such as the BBB, through which this crosstalk occurs. Currently, there are several tools available for the measurement of BBB permeability in humans, including dynamic contrast enhanced (DCE) MR imaging and blood–brain permeability imaging (BBPI), both of which quantify leakage of gadolinium contrast into the tissue parenchyma [158, 159]. Using DCE, BBB breakdown adjacent to the hippocampus has been demonstrated in individuals at risk for AD [160]. However, DCE and BBPI have not yet been employed to examine the effect of peripheral inflammation on BBB function. One limitation of DCE and BBPI is that they require the use of gadolinium as a contrast agent, a feature which may preclude the application of these imaging modalities to certain medically compromised patient groups. Proteins measured in CSF and blood have also been used to quantify BBB and blood-CSF barrier (BCSFB) permeability, including albumin (ratio of CSF albumin to serum albumin), IgG (ratio of CSF IgG to serum IgG), and fibrinogen [130]. Additionally, CSF levels of soluble platelet-derived growth factor receptor-ß (PDGFR-ß), a marker of pericyte injury, have been used to quantify BBB integrity [160], and more recently matrix metalloproteases 9 (MMP9) has been identified as potential markers of BBB dysfunction following SARS-CoV-2 infection [161]. These neuroimaging and fluid biomarkers have been employed to an extent in AD research, with recent studies suggesting that BBB integrity is likely affected early in the pathological cascade [160, 162, 163]. Although these studies raise important questions regarding the temporal sequencing of AD pathology deposition, immune dysregulation, and BBB integrity dysfunction, few studies have used these biomarkers to understand how acute inflammatory insults may affect neurobiological processes [164].

Understanding the pathways through which peripheral inflammation may interact with neurovasculature to influence AD risk will require the development and implementation of minimally invasive and scalable biomarkers of BBB integrity and endothelial function/activation. Ideally, these biomarkers would be inexpensive, suitable for use in longitudinal research, and safe enough to be applied in the setting of acute illness. With recent advances in proteomics, measurement of thousands of proteins in blood is now available at low cost, offering the potential for the identification of new blood-based BBB integrity and endothelial function biomarkers [24]. The ideal biomarker would be reliable, tissue-specific, possess a large dynamic range, and be validated against gold standard BBB/endothelial markers. The measurement of BBB/endothelial biomarkers alongside measures of neuroinflammation in individuals exposed to inflammatory insults will facilitate an improved understanding of the mechanisms through which peripheral inflammatory signaling influences neuro-immune activation and AD pathogenesis.

### Neuroprotective therapies in the context of acute inflammatory insults

Prompted largely by research demonstrating an elevation of inflammatory biomarkers in patients with AD dementia, reduced risk of AD among patients taking anti-inflammatory drugs for treatment of autoimmune inflammatory conditions [165, 166], and considerable evidence from genetic studies pointing to immune mechanisms in AD pathogenesis, anti-inflammatory therapies have been considered for treatment and prevention of AD. In fact, there is a long history of attempts to slow or prevent the progression of AD using therapies which target peripheral inflammation. Much of this work has come in the form of attempts to repurpose non-steroidal anti-inflammatory drugs (NSAIDs) such as naproxen, ibuprofen, rofecoxib, and indomethacin [167–171]. None of these therapies have demonstrated efficacy in slowing or preventing AD. However, a number of therapeutic agents which target the peripheral and central immune/inflammatory response are currently in the experimental pipeline, representing 21%, 13%, and 5% of experimental drugs for AD in phase 1, 2, and 3 as of 2021 [172]. One drug currently undergoing a phase 2 trial is InmuneBio’s Xpro1595, which selectively inhibits soluble TNF (sTNF) without blocking signaling of trans-membrane TNF currently targeted by approved pharmacotherapies. By selectively targeting sTNF – the “bad” form of TNF which is believed to be a primary regulator of peripheral inflammation – Xpro1595 is expected to reduce risk for AD. Preclinical studies indicate that Xpro1595 can lower amyloid, improve cognition, and normalize the immune response in older 5xFAD mice with Alzheimer’s-like pathology already present [173]. Recently, a small phase 1b clinical trial of patients with Alzheimer’s dementia found that 12-week administration of Xpro1595 lead to declines in CSF pro-inflammatory proteins and improvement in MRI-defined white matter quality. Other new or repurposed drugs which target peripheral inflammation are also being tested. For example, lenalidomide (brand name: Revlimid), an anti-cancer drug with anti-inflammatory and immunomodulatory effects, is currently in a phase 2 trial for patients with MCI due to AD [174]. JNJ-40346527 (Edicotinib), a selective inhibitor of colony-stimulating factor-1 receptor (CSF-1R) tyrosine kinase that was originally evaluated for arthritis, inflammatory bowel disease, and cancer is being tested in a phase 1 trial in patients with MCI. Though this drug is known to have effects on peripheral immunity, it has also been shown to inhibit microglial proliferation and production of pro-inflammatory cytokines [175]. While these and other efforts have thus far attempted to reduce AD risk via long-term modulation of the immune response, less is known about how *acute* inflammatory events may be targeted pharmacologically to prevent or reduce risk of AD or other long-term neurocognitive sequelae.

In the setting of an acute stressor – pathogen exposure or tissue damage – inflammation can be a double-edged sword, as both an exaggerated and overly attenuated inflammatory response can be detrimental to the host. Strategies to dampen overall inflammation, especially in the perioperative and in-hospital setting, have resulted in little to no protective effects against complications such as delirium and cognitive decline. Further characterization of the molecular underpinnings of these complications are needed to develop and more specifically target therapeutics. At the preclinical level, a major conceptual shift has taken place in the last decade, whereby the resolution of acute inflammation – long considered to be a mere process of passive dilution of inflammatory mediators – has been uncovered as a programmed response that triggers the formation of mediators with potent pro-resolving and anti-inflammatory capacity [176]. Harnessing specific pro-resolving pathways in the context of acute inflammatory insults may provide neuroprotective effects, particularly for vulnerable individuals.

Several specialized pro-resolving lipid mediators (resolvins) are synthesized as precursors to polyunsaturated fatty acids, including eicosapentaenoic acid (EPA) and docosahexaenoic acid (DHA). These lipid mediators are enzymatically produced throughout the body and have both anti-inflammatory and pro-resolving properties [176, 177]. Resolvins act through G-protein coupled receptors on immune, glia, and neuronal cells and can inhibit MAPK, NF-kB, and PI3K/Akt signaling pathways involved in pro-inflammatory cytokine production [178, 179]. These specialized pro-resolving lipid mediators are especially well-suited to limit excessive and potentially harmful inflammation following acute immune stressors. To date, there is evidence from animal models of surgery indicating that resolvins can limit peripheral inflammation, neuroinflammation, and post-operative cognitive decline [133, 180]. Furthermore, in AD rodent models, resolvins have been found to limit microglial pro-inflammatory signaling while also enhancing Aß phagocytic capacity [181, 182]. For example, using an APP AD mouse model, one study recently demonstrated that nasal administration of a combination of multiple resolvins (E1, RvD1, RvD2, Maresin 1, and neuroprotectin D1) decreased memory deficits and microglial activation, as indicated by a reduction in Iba1-posotive microglia [183]. Similarly, treatment of a Parkinson’s disease rat model with resolvin RvD1 has been shown to reduce microgliosis in the substantia nigra pars compacta and INF-γ level in the CSF [184].

Despite these encouraging findings, there is now evidence that human immune cells have low biosynthetic capacity for pro-resolving lipid mediators, making quantification difficult and challenging the idea that endogenous pro-resolving lipid mediators play a central role in inflammation resolution [185]. Notably, another endogenous pro-resolving mediator, Annexin A1 (ANXA1), is currently being investigated as a potential therapy In the context of AD [186]. As an effector of anti-inflammatory glucocorticoid signaling, ANXA1 has demonstrated an ability to suppress leukocyte inflammatory processes [187], limit microglial activation, improve microglial phagocytosis [188], restore BBB function and reduce tau phosphorylation in an AD rodent model [186]. Early phase human studies examining the safety and efficacy of pro-resolving molecules are now needed.

### Understanding the biological heterogeneity of acute inflammatory insults

Although we have framed our discussions of these acute inflammatory insults as complementary or related risk factors for AD, a provocative and important consideration is whether infectious and non-infectious events can be considered biologically similar in their effects on AD pathogenesis outside of the acute and relatively time-limited nature of their exposures. Whereas data linking acute infections to negative long-term CNS outcomes are growing and the direction of the results is fairly consistent, the associations between non-infectious acute events, particularly surgical procedures, and the same prospective outcomes are less clear. This raises several questions regarding the short- and long-term pathological cascades of these events, as well as the biological heterogeneity of and potentially differential risks for AD following acute infectious or non-infectious etiologies. Information gleaned from the proposed roadmap should provide a more granular understanding of how exposures to different acute inflammatory events influence or alter aging trajectories in mid- to late-life and should further reconcile whether—and how—potentially heterogeneous biological pathways converge on the pathogenesis of AD. The net result of these studies may be discrete conceptual frameworks for how acute infectious and non-infectious insults confer AD risk, despite similarities in the duration of their exposures.

## Conclusions

Mounting evidence suggests that acute inflammatory insults may have a deleterious effect on late-life cognition and increase risk for development of AD and related dementias. However, our understanding of potential mechanistic pathways through which acute inflammatory events may influence brain health is still hampered by methodological limitations, gaps in multidisciplinary and multi-modal research, suboptimal study design, and a limited number of longitudinal research efforts. Herein, we have proposed a roadmap to address these barriers to progress, and in doing so we have highlighted the need for interdisciplinary appraisal of both pathogen- and damage-mediated inflammatory insults across the lifespan and scalable biomarkers which capture the integrity, function, and activity of key periphery-to-CNS conduits. Moreover, we have provided recommendations for neuroprotective intervention studies focused on resolving inflammation following acute insults in an effort to reduce risk for subsequent cognitive decline and dementia. We anticipate that these efforts will advance our understanding of the role of the immune system in AD pathogenesis and eventually arm clinicians with the knowledge and tools needed to preserve brain health following acute inflammatory insults.

## Supplementary Information

**Additional file 1.**

## Data Availability

Not applicable.

## Abbreviations

Aβ: Amyloid-beta
AD: Alzheimer’s disease
BBB: Blood–brain barrier
BBPI: Blood–brain permeability imaging
CNS: Central nervous system
CRP: C-reactive protein
CVO: Circumventricular organ
DAMP: Damage associated molecular pattern
DCE: Dynamic contrast enhanced
DHA: Docosahexaenoic acid
EC: Endothelial cell
EPA: Eicosapentaenoic acid
ICU: Intensive care unit
LPS: Lipopolysaccharide
MCI: Mild cognitive impairment
NSAIDs: Non-steroidal anti-inflammatory drugs
NVU: Neurovascular unit
PDGFR-ß: Platelet-derived growth factor receptor-ß
POCD: Post-operative cognitive decline
TJ: Tight junction
TLR: Toll-like receptor
UTI: Urinary tract infection
