# Supplementary material for: The role of peripheral inflammatory insults in Alzheimer’s disease: a review and research roadmap
Source: Mol Neurodegener. 2023 Jun 5;18:37. doi: 10.1186/s13024-023-00627-2 (PMC10240487; doi:10.1186/s13024-023-00627-2)
Supplement: Supplementary file 1 — Additional file 1. [file 13024_2023_627_MOESM1_ESM.docx]

**Supplementary Table 1**. Studies examining the association between specific infections and Alzheimer’s disease, vascular dementia, all-cause dementia, and cognition

| **Author, Year, Doi** | **Cohort/Patient Registry or Country**  **(Total sample size)** | **Infection Type** | **Infection Diagnostic Method** | **Outcome**  **(Follow-up years*)** | **Results** |
| --- | --- | --- | --- | --- | --- |
| **Herpes Viruses** | | | | | |
| Allnut et al.,2020, 10.1016/j.neuron.2019.12.031 | MSBB  (n=301)  ROSMAP  (n=600) | HHV-6A  HHV-6B  (present/absent) | Brain tissue RNA-seq with PathSeq and ddPCR | AD (post-mortem) | HHV-6A: --AD  HHV-6B: --AD |
| Bae et al., 2021, 10.1007/s00406-020-01157-4 | HIRA-NSC  (n=229,594) | VZV  Antiviral medication  (present/absent) | EHR | ACD, AD, VaD (11) | VZV: ↑ACD, ↑AD, ↑VaD  Antivirals: ↓ACD |
| Choi et al., 2021 ,  10.1177/15333175211006504 | HIRA-NSC  (n=57,225) | VZV  (present/absent) | EHR | AD (11) | VZV: --AD |
| Chen et al., 2018,  10.4088/JCP.16m11312. | NHI  (n=78,410) | VZV  Antiviral medication  (present/absent) | EHR | ACD (16) | VZV: ↑ACD  Antivirals: ↓ACD |
| Itzhaki et al., 1997, 10.1016/S0140-6736(96)10149-5 | United Kingdom (England)  (n=90) | HSV1  (present/absent) | Brain tissue PCR | AD (post-mortem) | HSV1: ↑AD in *APOE4* carriers |
| Letenneur et al., 2008, 10.1371/journal.pone.0003637 | Personnes Agees QUID (n=512) | HSV1  (present/absent) | Serum IgGs and IgMs | AD (14) | IgM: ↑AD  IgG: --AD |
| Linard et al., 2019, 10.1002/alz.12008 | Three-City study (n=1,037) | HSV1  (present/absent; titers) | Serum IgGs and IgMs | ACD, AD/mixed dem. (10) | IgG or level: --ACD  IgG or level: --AD/mixed dem.  IgG+IGM: --ACD  IgG+IGM: --AD/mixed dem.  IgG (upper tercile)+IGM: --ACD in *APOE4* carriers  IgG (upper tercile)+IGM: ↑ AD/mixed dem. in *APOE4* carriers |
| Lindman et al., 2020,  10.1002/trc2.12119 | NPR  (n=265,172) | HSV1/2  VZV  Antiviral medication  (present/absent) | EHR | ACD (12) | HSV1/2 or VZV: ↑ACD  Antivirals: ↓ACD |
| Lovheim et al., 2015,  10.1016/j.jalz.2014.07.157 | Northern Sweden Health and Disease Study  (n=720) | HSV1/2  (present/absent; titers) | Plasma IgGs and IgMs | AD (15) | IgG: --AD  IgG level: --AD  IgM: --AD  IgG > 6.6 yrs. before dx: ↑AD  IgM > 6.6 yrs. before dx: -- AD |
| Lovheim et al., 2015,  10.1016/j.jalz.2014.04.522 | Betula study  (n=3,432) | HSV1/2  (present/absent) | Serum IgGs and IgMs | AD (20) | IgG: --AD  IgM: ↑AD |
| Readhead et al., 2018,  10.1016/j.neuron.2018.05.023 | MSBB  (n>100)  ROSMAP  (n=598)  Mayo Clinic  (n=298) | 515 viruses  (copy numbers) | Brain tissue RNA-seq with Viromescan | AD (post-mortem) | HHV6A: ↑AD  HHV7: ↑AD  HSV1: ↑AD |
| Schnier et al., 2021,  10.1111/ene.14795 | SAIL  (n=434,689)  Disease Analyzer (n=53,629)  DNR  (n=1,712,100)  eDRIS (n=342,637) | HSV  VZV  Antiviral medication  (present/absent) | EHR + HDR | ACD  AD  VaD (25) | Any herpes: ↑ACD, ↑AD  VZV: ↑ACD  Antivirals: ↓ACD |
| Tsai et al., 2017,  10.1371/journal.pone.0188490 | NHI  (n=3,384) | VZV  (present/absent) | EHR | ACD (5) | VZV: ↑AD |
| Tzeng et al., 2018,  10.1007/s13311-018-0611-x | NHI  (n=33,448) | HSV1  HSV2  Antiviral medication  (present/absent) | EHR | ACD, AD, VaD, Other dem. (11) | HSV1: ↑ACD, ↑AD, ↑VaD, ↑Other dem.  HSV2: --ACD, --AD, --VaD, --Other dem.  Antivirals: ↓ACD, ↓AD, ↓VaD, ↓Other dem. |
| Warren-Gash et al., 2022, 10.1002/acn3.51525 | CPRD  (n=884,045) | VZV  (present/absent) | EHR | ACD (18) | VZV: --ACD |
| Warren-Gash et al., 2019, 10.1038/s41598-019-41218-w | Meta-analyses  (n=not reported; 57 studies) | HSV1  HSV2  HHV6  CMV  EBV  (present/absent) | EHR, serum IgGs and IgMs, brain tissue PCR | AD (not reported) | HSV1: --AD  HSV2: --AD  HHV6: --AD  CMV: --AD  EBV: --AD |
| Stebbins et al., 2020,  10.1093/aje/kwaa238 | HRS  (n=5,617) | CMV  (absent/present; titers) | Serum IgGs | Global cognition (cross-sectional) | CMV: --Global cognition  CMV (levels): --Global cognition |
| Lovheim et al., 2018, 10.3233/JAD-161305 | Northern Sweden Health and Disease Study  (n=720) | CMV  HSV1  HSV2  (present/absent) | Plasma IgGs and IgM | AD (15) | CMV IgG: --AD  CMV IgM: --AD  HSV1 IgG: --AD  HSV2 IgG: --AD  HSV IgM: --AD  IgG CMV + IgG HSV1: ↑AD  IgG CMV + IgG HSV2: --AD |
| Barnes et al., 2015, 10.1093/infdis/jiu437 | ROSMAP and MARS (n=849) | CMV  HSV1  (present/absent) | Serum IgGs | AD, Global cognition (5) | CMV: ↑AD  CMV: ↓Global cognition  HSV1: --AD  HSV1: --Global cognition |
| Lee et al., 2020,  10.1186/s12883-020-01776-3 | HIRA-NSC  (n=4,122) | CMV  (present/absent) | EHR | ACD  AD  VaD  Other dem. (7) | CMV: ↑ACD  CMV: ↑AD  CMV: ↑VaD  CMV: ↑Other dem. |
| Dickerson et al., 2003,  10.1001/archpsyc.60.5.466 | USA  (n=229) | HSV1  HSV2  CMV  EBV  HHV6  VZV  (present/absent) | Serum IgGs | Global cognition (cross-sectional) | HSV1: ↓Global cognition  HSV2: --Global cognition  CMV: --Global cognition  EBV: --Global cognition  HHV6: --Global cognition  VZV: --Global cognition |
| Nimgaonkar et al., 2016, 10.1097/WAD.0000000000000133 | Monongahela–Youghiogheny Healthy Aging Team  (n=1,022) | HSV1  HSV2  CMV  *Toxoplasma gondii*  (present/absent) | Serum IgGs | Attention  Executive function Memory  Language  Visuospatial function  MMSE (5) | HSV1: --Attention, --executive function, --memory,  --language, --visuospatial function, --MMSE  HSV2: ↓Memory  HSV2: --Attention, --executive function, --language, --visuospatial function, --MMSE  CMV: ↓Memory, ↓visuospatial function  CMV: --Attention, executive function, language, MMSE  *T. gondii*: ↓Executive function, ↓MMSE  *T. gondii*: --Attention, --memory,  --language, --visuospatial function |
| Aiello et al., 2006,  10.1111/j.1532-5415.2006.00796.x | Sacramento Area Latino Study on  Aging  (n=1,204) | CMV  HSV1  (titers) | Serum IgGs | MMSE  Episodic memory  (4) | CMV: ↓MMSE  CMV: --Episodic memory  HSV1: --MMSE, --episodic memory |
| **Pneumonia** | | | | | |
| Shah et al., 2013,  10.1164/rccm.201212-2154OC | CHS  (n=5,888) | Hospitalized pneumonia, sepsis, ‘other’ infections  (present/absent) | EHR | ACD, Global cognition (10) | Pneumonia, sepsis, ‘other’ infections: ↑ACD  Pneumonia, sepsis, ‘other’ infections: ↓Global cognition |
| Chiu et al., 2022,  10.1016/j.bbi.2022.04.002 | NHI  (n=11,712) | Hospitalized bacterial pneumonia, subtypes (Streptococcus, Staphylococcus, Pseudomonas, Klebsiella, Hemophilus, Mycoplasma, Other gram-negative bacteria  (present/absent; frequency; admitted days) | EHR | ACD  AD  VaD  Other dem. (17) | Any bacterial pneumonia, Hemophilus: ↑ACD, ↑AD, ↑VaD, ↑Other dem.  Streptococcus, Staphylococcus, Pseudomonas, Klebsiella: ↑ACD, ↑VaD, ↑Other dem.  Mycoplasma: ↑ACD, ↑Other dem.  Other gram-negative bacteria: ↑ACD, ↑AD, ↑VaD  Pneumonia frequency, admitted days: ↑ACD, ↑VaD, ↑Other dem. |
| Davydow et al., 2013,  10.1016/j.amjmed.2012.12.006 | HRS  (n= 1,434) | Hospitalized pneumonia  (present/absent) | Medicare claims | Cognitive impairment (13) | Pneumonia: ↑Cognitive impairment |
| Tate et al., 2014,  10.1097/CCM.0000000000000123 | GEM  (n=3,069) | ICU pneumonia  (present/absent) | HDR | ACD (9) | Pneumonia: ↑ACD |
| Gerard et al,. 2006,  10.1111/j.1574-695X.2006.00154.x | Michigan AD Research Center, Canadian and UCLA Brain Tissue Banks,  Regions Hospital AD Research Center  (n=54) | *Chlamydia pneumoniae*  (present/absent) | Brain tissue PCR | AD (post-mortem) | *C. pneumoniae*: ↑AD |
| Balin et al., 1998,  10.1007/s004300050071 | Harvard Brain Tissue  Resource Center  (n=38) | *Chlamydia pneumoniae*  (present/absent) | Brain tissue PCR + IHC | AD (post-mortem) | *C. pneumoniae*: ↑AD |
| **Sepsis** | | | | | |
| Guerra et al., 2012,  10.1186/cc11901 | USA  (n=25,368) | ICU infection, sepsis  (present/absent) | Medicare claims | ACD (3) | Infection: ↑ACD  Sepsis: ↑ACD |
| Ahlstrom et al., 2020,  10.1186/s13054-020-03203-y | NPR  (n=210,334) | ICU sepsis  (present/absent) | EHR | ACD (11) | Sepsis: --ACD |
| Iwashyna et al., 2010,  10.1001/jama.2010.1553 | HRS (n=5,033) | Sepsis  (present/absent) | Medicare claims | Cognitive impairment (8) | Sepsis: ↑Cognitive impairment |
| Sakusic et al., 2018,  10.1097/CCM.0000000000003395 | Mayo Clinic  (n= 98,227) | ICU sepsis  (present/absent) | EHR | Cognitive impairment (2) | Sepsis: --Cognitive impairment |
| Lei et al., 2022,  10.3389/fnagi.2022.839472 | Meta-analyses (n=891,562; 8 Studies) | Sepsis  Severe sepsis  (present/absent) | EHR | ACD (11)  Cognitive impairment (8) | Sepsis: ↑ACD  Sepsis: --Cognitive impairment  Severe sepsis: ↑ACD  Severe sepsis: ↑Cognitive impairment |
| Fritze et al., 2021,  10.1212/NXI.0000000000000911 | Germany  (n=161,567) | Sepsis  Severe sepsis  (present/absent) | Insurance claims | ACD (11) | Sepsis: ↑ACD  Severe sepsis: ↑ACD |
| Kao et al., 2015,  10.1016/j.jocn.2015.02.035 | NHI  (n=11,910) | Sepsis  (present/absent) | EHR | ACD (9) | Sepsis: ↑ACD |
| **Periodontitis** | | | | | |
| Dominy et al., 2019, 10.1126/sciadv.aau333 | New Zealand Brain Bank  (n=58) | *Porphyromonas gingivalis*  (present/absent) | Brain tissue IHC | AD (post-mortem) | *P. gingivalis*: ↑AD |
| Ide et al., 2016,  10.1371/journal.pone.0151081 | United Kingdom (England)  (n=59) | *Porphyromonas gingivalis*  (present/absent; titers) | Serum IgGs | ADAS-cog, sMMSE (0.5) | *P. gingivalis*: ↓ADAS-cog  *P. gingivalis*: --sMMSE  *P. gingivalis* (level): --ADAS-cog  *P. gingivalis* (level): --sMMSE |
| Noble et al., 2009, 10.1136/jnnp.2009.174029 | NHNES III  (n = 2,355) | *Porphyromonas gingivalis*  (titers) | Serum IgGs | Delayed verbal memory  Immediate verbal memory  Serial subtraction  (cross-sectional) | *P. gingivalis* (upper quartile): ↓Delayed verbal memory  *P. gingivalis* (upper quartile):  --Immediate verbal memory  *P. gingivalis* (upper quartile): ↓Serial subtraction |
| Panzarella et al., 2022,  10.3233/JAD-200385 | Zabut Aging Project  (n=60) | *Aggregatibacter actinomycetemcomitans*, *Fusobacterium*  *nucleatum*, *Porphyromonas gingivalis*,  *Prevotella intermedia*,  *Treponema denticola*,  *Tannerella forsythia* (titers) | Mucosa PCR | AD (10) | *A. actinomycetemcomitans*: --AD  *F.* *nucleatum*: ↑AD  *P. gingivalis*: --AD  *P. intermedia*: --AD  *T. denticola*: --AD  *T. forsythia*: --AD |
| Chen et al., 2017, 10.1186/s13195-017-0282-6 | NHI  (n=27,963) | Chronic periodontitis  (present/absent) | EHR | AD (8) | Chronic periodontitis: --AD  Chronic periodontitis (>10yrs): ↑AD |
| Beydoun, et al., 2022, | NHNES III  (n=6,650) | 19 periodontal  pathogens (titers) | Serum IgGs | ACD  AD (26) | *C. rectus*: ↑ACD  *T. Denticola*: ↑ACD  *A. Naeslundii*: ↓AD  *C. rectus*: --AD  *T. Denticola*: --AD  *A. Naeslundii*: *--*ACD  *P. gingivalis*, *P. Intermedia*, *P. Nigrescens*, *T. Forsythia*, *A. Actinomycetemcomitans*, *F. Nucleatum*, *S. Oralis*, *M. Micros*, *E. Corrodens*, *E. Nodatum*, *S. Intermedius*, *C. Ochracea*, *V. Parvula*, *P. Melaninogenica*, *S. Noxia*, *S. Mutans*: --ACD, --AD |
| **Other infections (viruses, bacteria and fungi)** | | | | | |
| Imfeld et al., 2016,  10.1016/j.bbi.2016.03.014 | CPRD  (n=48,062) | Influenza  (present/absent; frequency) | EHR | AD (15.5) | Influenza: --AD  Frequency: --AD |
| Tseng et al., 2015,  10.1007/s10096-014-2200-1 | NHI  (n=86,182) | Chronic  Osteomyelitis  (present/absent) | EHR | ACD (12) | Osteomyelitis: ↑AD |
| Maheshwari & Eslick, 2015, 10.3233/JAD-140621 | Meta-analyses (n=1,204; 25 Studies) | Spirochetal bacteria *Chlamydophila pneumoniae*  (present/absent) | Brain tissue PCR + IHC, serum IgGs | AD (post-mortem; not reported) | Spirochetes: ↑AD  *C. pneumoniae*: ↑AD |
| Alonso et al., 2018,  10.3389/fnagi.2018.00159 | NDRC  (n=24)  Netherlands Brain Bank (n=3) | *Alternaria*,  *Aspergillus*,  *Botrytis*,  *Candida*, *Cladosporium*,  *Davidiella, Malassezia*,  *Phoma*  (present/absent) | Brain tissue PCR | AD (post-mortem) | *Alternaria*: ↑AD  *Aspergillus*: ↓AD  *Botrytis*: ↓AD  *Candida*: ↓AD  *Cladosporium*: ↑AD  *Davidiella*: ↓AD  *Malassezia*: ↑AD  *Phoma*: ↓AD |
| Pisa et al., 2017,  10.1038/s41598-017-05903-y | NDRC (n=18) | *Borrelia burgdorferi*  *Candida albicans*  *Chlamydophila pneumoniae*  *Clostridium*  (present/absent) | Brain tissue IHC | AD (post-mortem) | *B. burgdorferi*: ↑AD  *C. albicans*: ↑AD  *C. pneumoniae*: ↑AD  *Clostridium*: ↑AD |
| Pisa et al., 2015,  10.3233/JAD-141386 | NDRC (n=6) | *Candida glabrata*  (present/absent) | Brain tissue IHC | AD (post-mortem) | *C. glabrata*: ↑AD |
| Beydoun, et al., 2018,  10.1016/j.jalz.2018.04.009 | NHNES III  (n=5,927) | *Helicobacter pylori*  (present/absent) | Serum IgGs | ACD  AD (26) | *H. pylori*: ↑ACD  *H. pylori*: ↑AD |
| Miklossy., 1993,  (DOI NA) | Switzerland  (n=27) | *Borrelia burgdorferi*  (present/absent) | CSF microscopy | AD (post-mortem) | *B. burgdorferi*: --AD |
| Marques et al., 2000,  10.1086/315792 | Hopkins AD Research Center  (n=30) | *Borrelia*  (present/absent) | Brain tissue PCR | AD (post-mortem) | *Borrelia*: --AD |
| Galbussera et al., 2008,  10.1097/WAD.0b013e31816ba95d | Italy  (n=98) | *Borrelia burgdorferi* | Serum IgGs and IgMs | AD (cross sectional) | *B. burgdorferi*: --AD |
| McLaughlin et al., 1999, 10.1097/00001756-199905140-00018 | Canada  (n=28) | Spirochetes | Plasma microscopy | AD (cross sectional) | Spirochetes: --AD |
| Zhan et al., 2016, 10.1212/WNL.0000000000003391 | AD Center, UC Davis  (n=42) | *Escherichia coli*  Lipopolysaccharide  (preset/absent; protein levels) | Brain tissue PCR + ICC | AD (post-mortem) | *E. coli*—AD  LPS--AD  *E. coli* (levels)↑AD  LPS (levels)↑AD |
| Chiu et al., 2014, 10.1111/ene.12317 | NHI  (n=117,140) | HCV  (present/absent) | EHR | ACD (13) | HCV: ↑ACD |
| Choi et al., 2021, 10.1097/MD.0000000000026476 | HIRA-NSC  (n=56,140) | HBV  HCV  (present/absent) | EHR | AD (12) | HBV: --AD  HCV: ↑AD |
| Lin et al., 2017,  10.1371/journal.pone.0179312 | NHI  (n=438) | HCV  (present/absent) | EHR | AD (14) | HCV: ↑AD |
| Lam et al., 2022, 10.1097/QAD.0000000000003134 | USA  (n=168,650) | HIV  (present/absent) | Insurance claims | ACD (17) | HIV: ↑ACD |
| Lam et al., 2021, 10.1097/QAD.0000000000002806 | USA  (n=124,403) | HIV  (present/absent) | Insurance claims | ACD (6) | HIV: ↑ACD |
| Bobrow et al., 2020, 10.1097/QAD.0000000000002597 | Veterans Health Administration (n=2,228) | HIV  (present/absent) | EHR | ACD (11) | HIV: ↑ACD |
| Goodkin et al., 2017,  10.1016/S2352-3018(17)30098-X | MACS  (n=5,086) | HIV  (Disease stage) | Plasma load + CD4^+^ count | Information processing speed  Executive function  Episodic memory  Working memory Motor function (32) | Disease stage: --Information processing speed  Disease stage: --Executive function  Disease stage: ↓Episodic memory  Disease stage: --Working memory Disease stage: ↓Motor function |
| Hampshire et al., 2021,  10.1016/j.eclinm.2021.101044w | United Kingdom  (n=81,337) | SARS-CoV-2  (present/absent) | Mucosa PCR or IgGs | Global cognition (1) | SARS-CoV-2: ↓Global cognition |
| Liu et al., 2021,  10.1186/s13024-021-00469-w | China  (n=1,539) | SARS-CoV-2  Severe SARS-CoV-2  (present/absent) | Mucosa PCR or IgGs | Global cognition (0.5) | SARS-CoV-2: --Global cognition  Severe SARS-CoV-2: ↓Global cognition |
| Wang et al., 2022,  10.3233/JAD-220717 | USA  (n=821,496) | SARS-CoV-2 | EHR | AD (1) | SARS-CoV-2: ↑AD |

↑ infection associated with increased risk/cognitive performance; ↓ infection associated with dementia risk/cognitive performance; -- null association. *Maximum follow-up time/observational window. Abbreviations: ACD, all-cause dementia; AD, Alzheimer’s disease; ADAS-cog, Alzheimer’s Disease Assessment Scale; aMCI, amnestic mild cognitive impairment; ARIC, Atherosclerosis Risk in Communities study; BKV, human polyomavirus BKV; CHS, Cardiovascular Health Study; CMV, cytomegalovirus; CPRD, Clinical Practice Research Datalink; DNR, Danish National Registries; EBV, Epstein–Barr virus; eDRIS, Electronic Data Research and Innovation Service; EHR, electronic health records; GEM, Gingko Effect on Memory study; HIRA-NSC, Korean Health Insurance Review and Assessment Service-National Sample Cohort; HBV, hepatitis B virus; HCV, hepatitis C virus; HDR, hospital discharge records; HHV, human herpes virus; HIV, human immunodeficiency virus; HPV, human papillomavirus; HRS, Health and Retirement Study; HSV, herpes simplex virus; HTLV, human T-cell lymphotropic virus; ICC, immunocytochemistry; ICU, intensive care unit; IHC, immunohistochemistry; IgG, immunoglobulin G; IgM, immunoglobulin M; JCV, human polyomavirus JCV; MACS, Multi-Center AIDS Cohort Study; MCV, Merkel cell polyomavirus; NDRC, Neurological Disease Research Center, Spain; NHI, National Health Insurance, Taiwan; NHNES III, National Health and Nutrition Examination Survey III; NOMAS, Northern Manhattan Study; NPR, National Patient Registry, Sweden; MARS, Minority Aging Research Study; MSBB, Mt. Sinai Brian Bank; PCR, polymerase chain reaction; ROSMAP, Religious Orders Study/Rush Memory and Aging Project; SAIL, Secure Anonymized Information Linkage Databank; sMMSE, standardized Mini-Mental State Examination; VaD, vascular dementia; VZV, varicella zoster virus.
